# Automated Neuroanatomical Relation Extraction: A Linguistically Motivated Approach with a PVT Connectivity Graph Case Study

**DOI:** 10.3389/fninf.2016.00039

**Published:** 2016-09-21

**Authors:** Erinç Gökdeniz, Arzucan Özgür, Reşit Canbeyli

**Affiliations:** ^1^Department of Computer Engineering, Boğaziçi Universityİstanbul, Turkey; ^2^Department of Psychology, Boğaziçi Universityİstanbul, Turkey

**Keywords:** text mining, natural language processing, connectivity relation extraction, neuroinformatics, brain region connectivity graph, brain region dictionary, paraventricular nucleus of the thalamus, PVT

## Abstract

Identifying the relations among different regions of the brain is vital for a better understanding of how the brain functions. While a large number of studies have investigated the neuroanatomical and neurochemical connections among brain structures, their specific findings are found in publications scattered over a large number of years and different types of publications. Text mining techniques have provided the means to extract specific types of information from a large number of publications with the aim of presenting a larger, if not necessarily an exhaustive picture. By using natural language processing techniques, the present paper aims to identify connectivity relations among brain regions in general and relations relevant to the paraventricular nucleus of the thalamus (PVT) in particular. We introduce a linguistically motivated approach based on patterns defined over the constituency and dependency parse trees of sentences. Besides the presence of a relation between a pair of brain regions, the proposed method also identifies the directionality of the relation, which enables the creation and analysis of a directional brain region connectivity graph. The approach is evaluated over the manually annotated data sets of the WhiteText Project. In addition, as a case study, the method is applied to extract and analyze the connectivity graph of PVT, which is an important brain region that is considered to influence many functions ranging from arousal, motivation, and drug-seeking behavior to attention. The results of the PVT connectivity graph show that PVT may be a new target of research in mood assessment.

## Introduction

Many studies have been conducted to identify the relations among brain regions in various species and this information is already available in the free text of the biomedical literature, albeit scattered in a large number of studies published over a sizable time period. Our aim is to propose a linguistically empowered approach by using natural language processing (NLP) techniques to automatically extract connectivity relations among brain regions from publications. By doing so, we target with the present study to obtain neuroanatomical connectivity among brain structures to be extended in subsequent studies to neurochemical and functional relations. After generating a map of connections, we will be in a position to automatically extract a brain region's relations and its effects on many functions such as arousal, motivation, depression and attention. As a case study, we focus on a specific brain region, the paraventricular nucleus of the thalamus (PVT), which belongs to midline and intralaminar group of thalamic nuclei and is long considered to have a non-specific effect on cortical arousal. Our main reason for choosing PVT as a particular target of research is that recent studies have begun to attribute more specific functions to this group of thalamic nuclei because of their rich neuroanatomical and neurochemical projections (Hsu and Price, 2009; Li and Kirouac, 2012; Vertes et al., 2015).

Most previous studies on text mining in the biomedical domain have focused on extracting information about proteins and genes from scientific publications. Shared tasks such as BioCreative (Krallinger et al., 2008; Arighi et al., 2011) and BioNLP (Kim et al., 2009, 2011; Nédellec et al., 2013) have boosted research in this area. Both rule-based (Fukuda et al., 1998; Hur et al., 2009) and machine learning based methods (McDonald and Pereira, 2005; Hsu et al., 2008) have been proposed for identifying names of proteins/genes in scientific texts. Several approaches ranging from entity co-occurrence (Jelier et al., 2005; He et al., 2009) and pattern matching based methods (Blaschke and Valencia, 2002) to more complex NLP and/or machine learning based methods have been proposed for extracting the relations among proteins (Giuliano et al., 2006; Erkan et al., 2007; Fundel et al., 2007; Airola et al., 2008; Tikk et al., 2010; Quan et al., 2014).

Developing text mining methods in the neuroinformatics domain for identifying brain region entities and mining the neuroanatomical relations among them is a relatively new research topic, compared to the more widely studied areas of biomedical text mining focusing on genes, proteins, and diseases. Only a handful of studies have been conducted in neuroscience text mining so far, most of which adapt and extend the methods proposed in the well-studied area of protein-protein interaction extraction. In the context of the Neuroscholar system, which is one of the first studies tackling the use of advanced NLP methods for neuroscience data mining, Burns et al. (2008) extracted neuroanatomical information from tract-tracing experiments with an F-Measure of 79% on identifying the mentions of five types of neuroscience named entities related to tract-tracing-experiments. They used conditional random fields (CRF) with a feature set utilizing morphological, lexical, syntactic and semantic information on a manually annotated corpus of 1047 sentences from 21 documents. French et al. (2009) had a similar CRF based approach with a richer feature set and reported 92% precision and 86% recall on the task of identifying brain region mentions in text. In their extended study, French et al. (2012) have focused on the connectivity between the entities and applied co-occurrence based methods and kernel-based supervised machine learning methods, which have originally been proposed for extracting protein-protein interactions. Tikk et al. (2010) evaluated nine different kernel based methods on the task of protein-protein interaction extraction and later on their study became the base evaluation framework in different tasks such as drug-drug interaction extraction (Segura-Bedmar et al., 2011) and neuroanatomical relation extraction (French et al., 2012, 2015; Richardet et al., 2015). French et al. (2012) reached high recall and low precision with co-occurrence based methods, whereas following the framework of Tikk et al. (2010) with the shallow linguistic kernel they obtained 70.1% recall and 50.3% precision values. Recently, within the WhiteText project aiming at developing corpora and tools for extracting neuroanatomical connectivity statements from text, French et al. (2015) tested their approach on an enhanced corpus with new abstracts and obtained similar findings with a precision of 51% and recall of 67%. Richardet et al. (2015) built their research on this approach by improving the kernels with filters and lexical rules developed according to the sentence structures. The proposed filters are mostly applied in order to remove the unlikely brain region connections and the rules mainly depend on the surface structures of the sentences such as the locations of the brain regions in the sentences. Vasques et al. (2015) extended this work to find the targets of a seed in tractography projects.

In the present paper, we propose a NLP based approach for neuroanatomical relation extraction from neuroscience publications. Unlike most previous neuroanatomical relation extraction studies that aimed at utilizing supervised machine learning based methods originally proposed for protein-protein interaction extraction, we target developing a high-precision knowledge-based linguistically motivated approach specifically designed for the neuroscience domain. Different from the rule-based method proposed in Richardet et al. (2015), which only makes use of the surface structures of sentences; we utilize the parse tree analyses of sentences. Our approach is based on using predefined patterns for selecting the potential neuroanatomical connectivity relation describing sentences and leveraging the deeper syntactic analysis of the sentences, specifically the constituency and dependency parse trees, for identifying the related brain region entities. The brain region entities are identified and normalized by utilizing a brain region dictionary created in this study. We use the WhiteText corpus (French et al., 2012, 2015) consisting of abstracts to develop and evaluate our approach. In addition, we present our results on a manually annotated corpus of 14 full text articles relevant to a specific brain region (PVT). Finally, as a case study, we apply our method to extract neuroanatomical relations from articles in PubMed relevant to PVT. During the extraction of the relations, we also focus on the direction of the relations, which allows us contribute to the prior work with a directional connectivity graph between the brain regions. The compiled brain region dictionary and the manually annotated sentences in the full-text corpus are additional contributions of our work and are made publicly available for future neuroscience text mining studies.

## Materials and methods

### Data preparation

#### Corpus

Two different corpora were used in this research. The first is the WhiteText corpus that contains 3205 abstracts manually annotated for brain region mentions and the interactions among them (French et al., 2012, 2015). As done in the previous studies (French et al., 2012, 2015; Richardet et al., 2015), we only considered the relations that are described in a single sentence, and discarded the ones that span multiple sentences. We used the first version of the corpus (French et al., 2012) consisting of 1377 abstracts (containing 3097 connectivity relations) as our development set, and the 1828 abstracts (containing 2111 connectivity relations) added to the second version of the corpus (French et al., 2015) as our test set.

The second corpus that we compiled and used was PVT specific. A list of 558 PVT related publications is retrieved from PubMed by using the following query (on 14th of August, 2015).

“*(“paraventricular”[All Fields] AND (“thalamic”[All Fields] OR “thalamus”[All Fields]) AND (“nucleus”[All Fields] OR “nuclei”[All Fields]) NOT “hypothalamus”[All Fields] NOT “hypothalamic”[All Fields]) OR (“Paraventricular Thalamic Nucleus”[All Fields] OR “paraventricular nucleus of thalamus”[All Fields] OR “paraventricular nucleus of the thalamus”[All Fields] OR “paraventricular thalamus”[All Fields])”*

The PVT corpus is used in two different ways during the evaluation. The abstracts of 451 publications (for which the full text was not publicly available) and 107 publicly available full text publications constituted the first data set and provided the basis for our application on the PVT case study. Secondly, 14 of these full text papers were selected by neuroscience domain experts and fully annotated with brain region mentions and connectivity statements. These 14 papers were selected randomly from a set of publications, which were known to be PVT related and included review papers. As the annotation guideline, we applied three steps. First, all brain region entities mentioned in the articles were annotated without regard to connectivity. Then all types of relations including neuroanatomical, neurochemical, and functional connections were marked. Lastly, we identified and evaluated only the neuroanatomical relations at the sentence level, when the text specifically mentioned identifiable connectivity between brain structures. Table 1 shows sample sentences from the annotated PVT corpus. The PubMed IDs of the publications in the PVT corpus, as well as the sentences manually annotated for neuroanatomical connectivity relations of the 14 full text papers are available as Supplementary Materials^1^.

**Table 1:** **Sample sentences from the annotated PVT corpus**.

**Sentence**	**Brain Region 1**	**Direction**	**Brain Region 2**
These experiments confirm projections from Pa, Pt, and other midline nuclei to the amygdala.	Pa	→	Amygdala
These experiments confirm projections from Pa, Pt, and other midline nuclei to the amygdala.	Pt	→	Amygdala
In addition, we found that the aPVT was strongly innervated by the ventral subiculum but this projection largely did not involve the pPVT.	aPVT	←	Ventral subiculum
The paraventricular thalamus (PVT), a midline thalamic nucleus, receives dense innervations from lateral hypothalamic orexin neurons (Peyron et al., 1998; Kirouac et al., 2005) and is involved in the regulation of cognition, anxiety, emotionality and addiction behaviors (Huang et al., 2006; Li et al., 2009, 2010a,b, 2011).	PVT	←	Hypothalamic orexin neurons

The first two sentences are from Hsu and Price (2009), the third sentence is from Li et al. (2014), and the last sentence is from Choi et al. (2012).

#### Creation of a brain region dictionary

We used a dictionary-based approach to identify the brain region entities that participate in neuroanatomical relations and normalized their mentions to canonical (unique) names. We constructed a dictionary of brain regions including their acronyms and synonyms, where an acronym is the abbreviation of the brain region entity and a synonym is a similar word or phrase used for the same brain region entity in text. A portion of the created dictionary with sample entries is shown in Table 2.

**Table 2:** **Sample entities from the Brain Region Dictionary**.

**Brain Region**	**Acronyms**	**Synonyms**
Parietal lobe	PL	Parietal cortex, parietal region, lobus parietalis
Suprachiasmatic nucleus	SCN	Suprachiasmatic nuclei
Cingulate gyrus	CgG	Cingular gyrus, cingulate area, cingulate region, Gyri cinguli, Gyrus cinguli
Subthalamus	SbTh	Subthalamic region, ventral thalamus, thalamus ventralis
Parabrachial nucleus	–	Parabrachial nuclei, parabrachial
Paracentral nuclues	PC	Paracentral thalamic nucleus, nucleus paracentralis, paracentral nucleus of the thalamus, paracentral

During the dictionary creation step, we initially gathered a dictionary of 892 brain regions and 562 acronyms from the NeuroNames ontology (Bowden and Dubach, 2003) and NeuroLex (Larson and Martone, 2013), which is a dynamic lexicon of neuroscience concepts. We expanded this initial dictionary by including synonyms of brain regions by investigating a set of neuroscience publications and compiling the different usages of brain region mentions in the neuroscience literature. We considered brain region direction information such as anterior, posterior, ventral, dorsal, rostral, and caudal during the dictionary creation process. For instance, “anterior PVT” and “PVT” are listed as separate brain region entities in our dictionary.

The resulting enriched dictionary contains 3044 brain region entities with their synonyms and acronyms. The created brain region dictionary is made publicly available as Supplementary Materials for future text mining studies^2^.

### Neuroanatomical relation extraction

**Figure 1:**
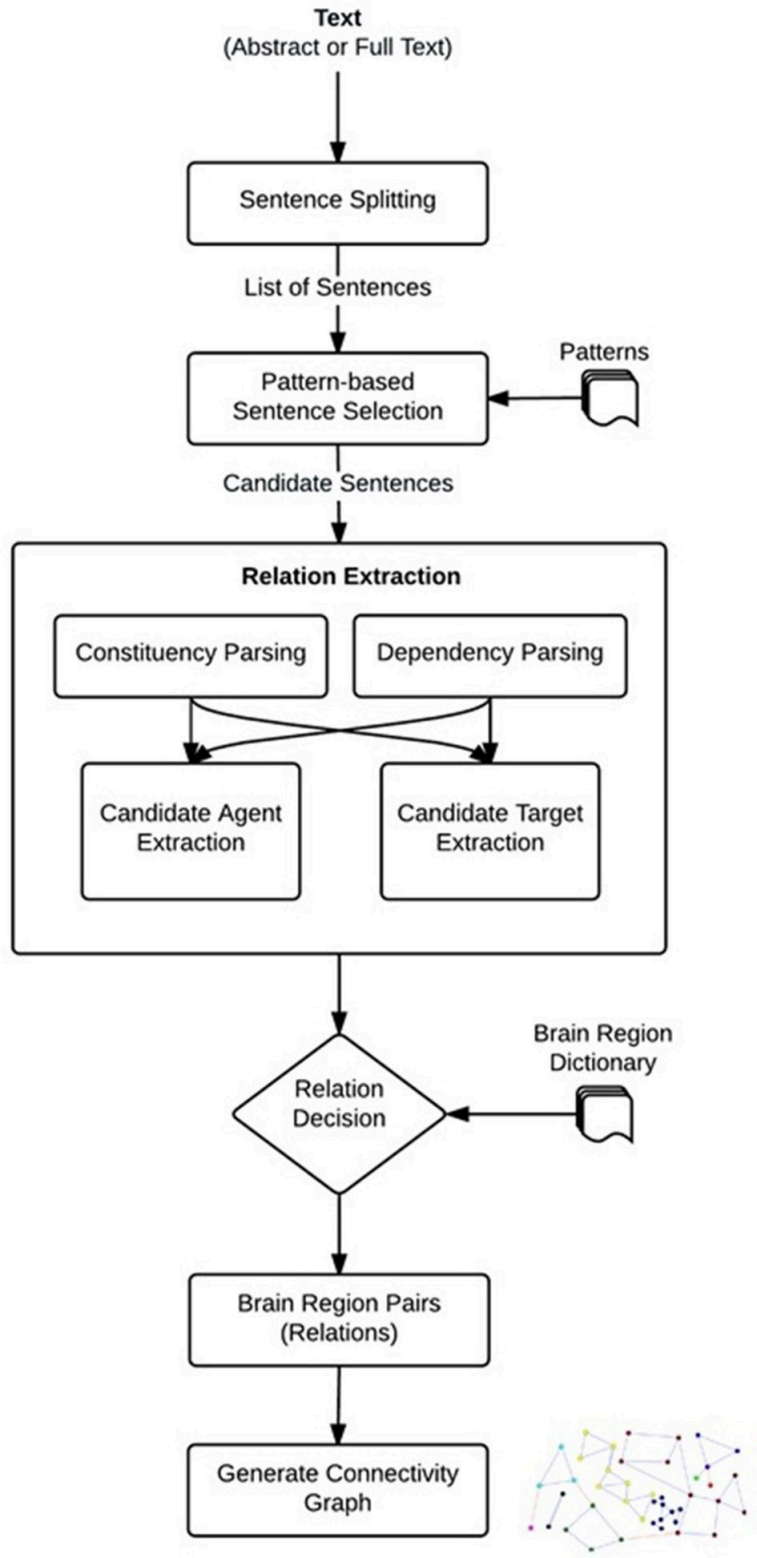
**Steps to extract neuroanatomical relations**.

We developed a linguistically motivated knowledge-based approach for neuroanatomical relation extraction. The workflow of the proposed approach is shown in Figure 1. Automated relation extraction in general relies on finding the correct sentences that describe an interaction between brain regions. For this purpose, as a first step, the publications (abstracts or full text) were split into sentences. After feeding these sentences into our system a list of candidate sentences, which might contain relations were selected by using predefined patterns. Then, NLP techniques were used to identify the brain regions that are described as being related in these sentences. We used the dependency and constituency parse trees of the sentences and applied linguistic rules over these parse trees to extract the portions of sentences that were likely to contain brain region entities participating in a neuroanatomical relation, i.e., the candidate brain region entities. Based on predefined patterns, we also identified relation directionality by labeling the candidate brain region entities as “agents” or “targets”. For example, from a sentence like “X receives input from Y,” we obtained the information that Y is the agent and X is the target of the relation, i.e., the directionality of the relation is Y → X. In the relation decision step, the candidate brain region entities were searched in the brain region dictionary, and a neuroanatomical relation was identified if the candidate agent and target were matched in the dictionary. Finally, the agents and targets of the identified neuroanatomical relations were normalized to their canonical names using the brain region dictionary and a directional brain region connectivity graph was created. The graph can be further analyzed to generate new scientific hypotheses. The details of each step in our method are described in the following sub-sections.

#### Sentence splitting

The Stanford Core NLP tool (Manning et al., 2014) was used for splitting the publications into their sentences.

#### Pattern-based sentence selection

After preparing the data, the first phase of relation extraction was to scan the publications and extract the sentences that contained the predefined patterns. The extracted sentences at this step were the first candidates that might include neuroanatomical relations among brain regions.

We manually designed a set of patterns, which are strings of keywords that mostly reveal a relation, when there are two or more brain region entities in a sentence. Some of the patterns that were used in this research are “projection to, innervation of, receive input from, project from, efferent from.” For example, the following sentence contains a relation between the “dorsal midline thalamus” and “accumbens nucleus” brain regions signaled by the pattern “projection to.”

“*An anterograde tracer injection into the **dorsal midline thalamus** revealed strong **projections to** the **accumbens nucleus***.” (Hsu and Price, 2009)

Neuroanatomical relations are in general signaled by pattern keywords. Since each keyword can have different prepositional suffixes (e.g., projection from, projection of, projection to) and different tenses (e.g., projects to, projecting to, projected to), regular expressions were used to cover the different usages of the patterns. As shown in the below regular expression for the pattern “project to,” the patterns were considered to be case insensitive and are likely to contain additional words between their original keywords (i.e., between “project” and “to”).

(?i)project(ing|s|ed) {0, 1} ((\w)^*^) {0, 2}to

The list of designed patterns and the corresponding regular expressions are shown in Table 3. The sentences in the publications that match these patterns (regular expressions) were selected as candidate sentences and provided as input to the relation extraction component described in the next sub-section.

**Table 3:** **List of the patterns that are used in the research**.

**List of Patterns**	
Innervate	(?i)innervat(e|es|ing){1}
innervation of	(?i)innervation(s){0,1} of
projection to	(?i)projection(s){0,1} to
projection to from	(?i)projection(s){0,1} to ((\\w+)\\s){0,8} from
projection of	(?i)projection(s){0,1} of
projection target of	(?i)projection target(s){0,1} of
projection from	(?i)projection(s){0,1} from
projection from to	(?i)projection(s){0,1} from ((\\w+)\\s){0,8} to
project to	(?i)project(ing|s|ed){0,1} ((\w)^*^){0,2}to
project into	(?i)project(ing|s|ed){0,1} ((\w)^*^){0,2}into
project from to	(?i)project(s|ed|ing){0,1} from ((\\w+)\\s){0,8} to
receive input from	(?i)receiv(e|es|ing|ed){0,1} ((\w)^*^){0,4}input(s){0,1} ((\w)^*^){0,3}(from)
receive fiber from	(?i)receiv(e|es|ing|ed){0,1} ((\w)^*^){0,4}fiber(s){0,1} ((\w)^*^){0,3}(from)
receive innervation from	(?i)receiv(e|es|ing|ed){0,1} ((\w)^*^){0,4}innervation(s){0,1} ((\w)^*^){0,3}(from)
receive [ae]fferent from	(?i)receiv(e|es|ing|ed){0,1} ((\w)^*^){0,4}[ae]fferent(s){0,1} ((\w)^*^){0,3}(from)
traveling from to	(?i)travel(s|ling){0,1} ((\w)^*^){0,2}from ((\w)^*^){0,5}to
exit through	(?i)exit(s|ing){0,1} ((\w)^*^)^*^through
exit from	(?i)exit(s|ing){0,1} ((\w)^*^)^*^from

#### Candidate generation using NLP techniques

After generating the list of sentences, which were candidates for hosting brain region relations, a detailed syntactic analysis of each sentence was done. There were two dependents of the patterns: agents and targets. If both of these dependents included brain region entities, then we considered that there was a relation between these entities. There could be more than one relation within a given sentence, if dependents included more than one brain region.

To be able to identify whether a dependent is an agent or target, we needed the directionality of the relation and this information was gathered directly from the patterns. For example, for the patterns like “receive input from, projection from, efferent from,” it is likely that the text string that follows the pattern is agent. On the other hand, for the “project into, innervate, terminate in” patterns, the same text reveals the target.

**Figure 2:**
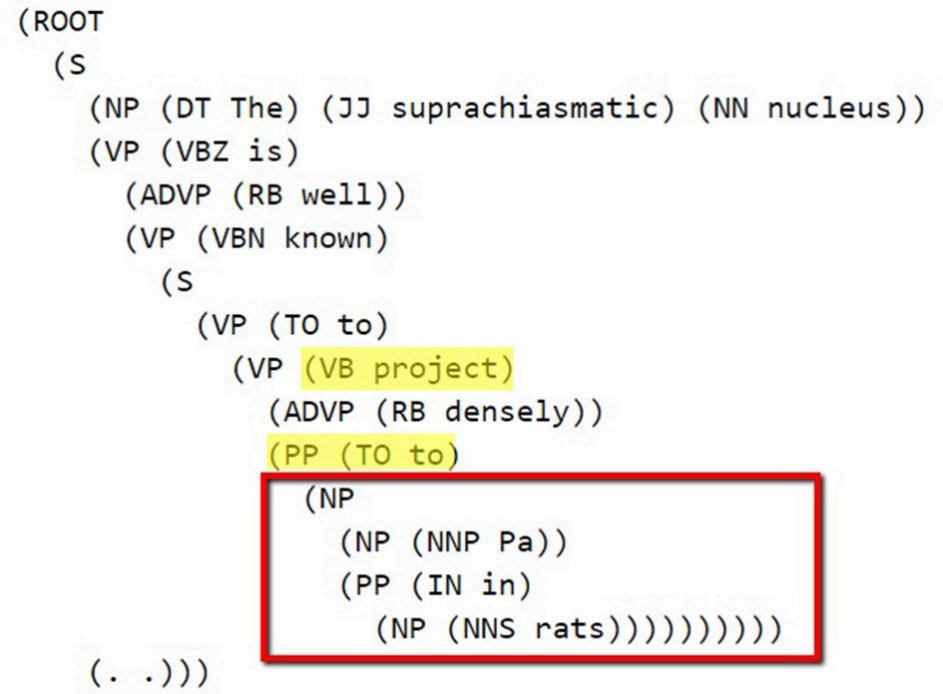
**Bracketed Notation of Parse Tree for the sentence: “*The suprachiasmatic nucleus is well known to project densely to Pa in rats.”*** The first noun phrase after the pattern (project to) is selected.

**Figure 3:**
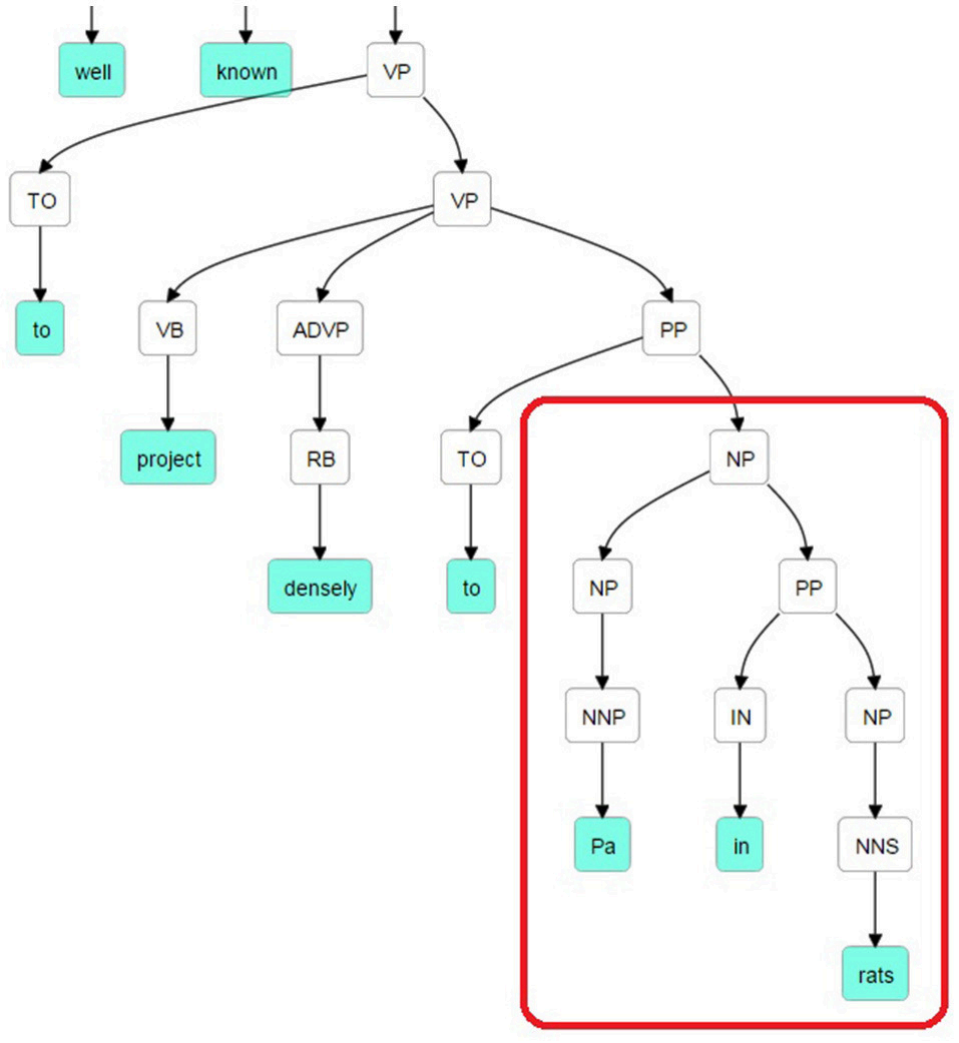
**Parse Tree for the sentence: “*The suprachiasmatic nucleus is well known to project densely to Pa in rats*.”** The retrieved candidate dependent is “Pa in rats.”

The Stanford Parser was used to syntactically parse the sentences and obtain their constituent elements (Klein and Manning, 2003). One of the dependents (agent or target) in general occurred right after the pattern keyword. The constituency (phrase structure) parse tree is traced until we reach the pattern and then we selected the first Noun Phrase (NP) following the pattern in the bracketed notation of the parse tree. After finding the NP, all of the leaves under this NP were used to generate the candidate dependent. In Figure 2, a bracketed notation of the parse tree for the “The suprachiasmatic nucleus is well known to project densely to Pa in rats” sentence (taken from Hsu and Price, 2009) is presented and in Figure 3 the tree representation of the same sentence is shown. The identified NP is enclosed in a box in these figures.

In some sentences, the prepositional phrase (PP) following the detected NP modifies the NP and may contain candidate dependents for the relation. Therefore, if a detected NP was followed by a PP, which contains the keyword “including,” then it was also added as part of the candidate brain region text (dependent). An example sentence is provided below.

“*Studies in rats show that the caudal DR projects strongly to limbic structures **including** the amygdala and hippocampus,…*” (Hsu and Price, 2009)

To find the first dependent (brain region candidate) that follows the pattern keyword, we used the constituency parser. On the other hand, for the second dependent, the text extraction phase was more complex. The second dependent can be found in different locations of the sentence. It can be at the beginning, right before the pattern, or close to the end of the sentence after the pattern. The dependency tree of a sentence can capture the long-distance relations among its words. We used the Stanford Dependency Parser (De Marneffe et al., 2006) to analyze the dependency structures of the sentences and obtain the second candidate dependent, which does not necessarily occur close to the pattern. The output of the Stanford Dependency Parser is the Stanford Dependencies representation, which is a description of the grammatical relationships among the words in a sentence (De Marneffe et al., 2006).

A dependency was considered as **relation(governor-pos**^1^**, dependent-pos**^2^**)** where the governor and the dependent were words in the sentence and pos^1^ and pos^2^ indicate the positions of the two words in the sentence. **Relation** is one of the 50 grammatical relations defined in the Stanford Parser (De Marneffe et al., 2006).

As the starting point of identifying the second dependent, when a pattern was found in a sentence, one of the dependency types below is searched in the dependency tree. The pattern keyword in these types could be either governor or dependent. The descriptions of all the dependency types can be found in the Stanford Parser dependencies manual with sample sentences and dependency trees^3^.

1. Direct object (dobj)
2. Nominal subject (nsubj)
3. Passive nominal subject (nsubjpass)
4. Controlling subject (xsubj)
5. Noun Compound Modifier (nn)
6. Reduced non-finite verbal modifier (vmod)

We worked on these grammatical relations under three different groups according to the sentence structures as described in the following sub-sections.

##### Relations where the pattern keyword is in nsubj/nsubjpass/xsubj/nn relations

This rule set was applied for the pattern keywords that contain *nsubj, nsubjpass, xsubj*, or *nn* type of relations. In these cases, the governor/dependent that was found in this relation was directly considered as a candidate brain region. Additionally, two different rules were applied when the pattern keyword was found in these relations.

**Figure 4:**
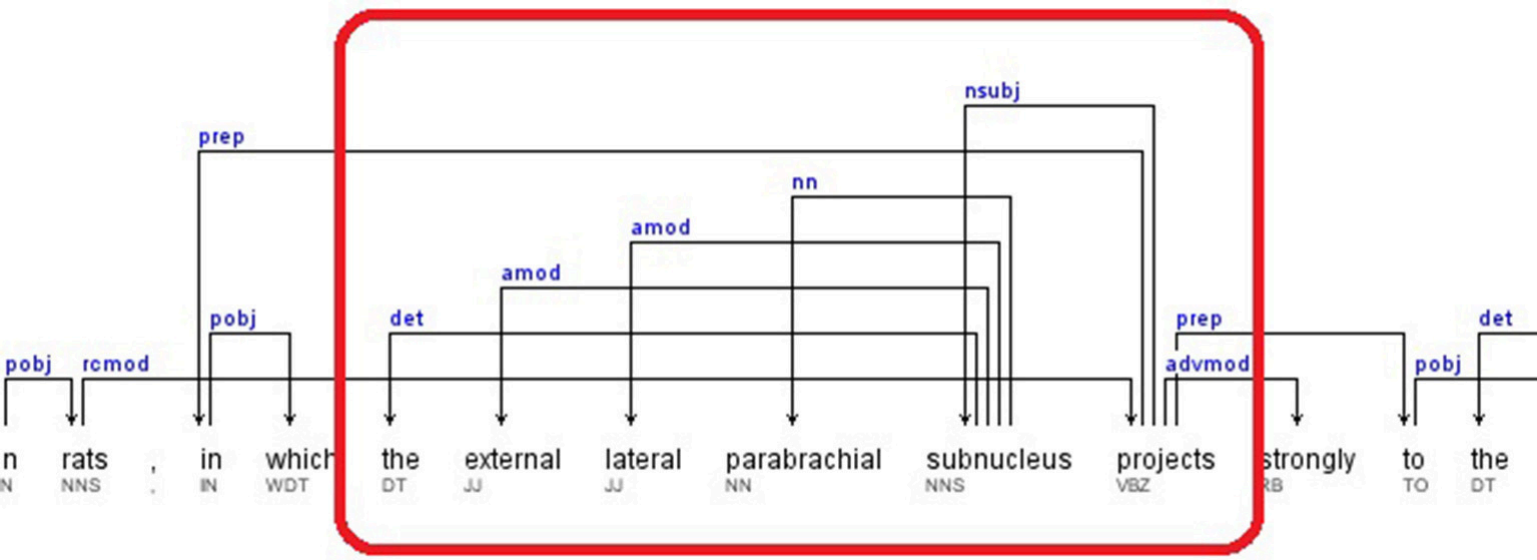
**Dependency Tree for the sentence: “*This topography is consistent with findings in rats, in which the external lateral parabrachial subnucleus projects strongly to the anterior paraventricular thalamic nucleus (Pa), and less so to the middle and posterior paraventricular thalamic nucleus (Pa)(Krout and Loewy, 2000)*”**.

1. If the pattern keyword was found as a dependent, then the Prepositional Modifier (*prep*) of the governor was retrieved. The dependent of the *prep* relation was selected as a candidate brain region. Then the Adjectival Modifier (*amod*) or Noun Compound Modifier (*nn*) relations are also gathered as parts of the candidate brain region.
2. If the pattern keyword was found as a governor, all the relations that contained the dependent as a governor were selected. The dependents of these relations are retrieved as candidate brain regions. A portion of the dependency tree for a sample sentence, for which this rule applies, is presented in Figure 4. The extracted relations and candidate brain regions from this sentence are presented below.

Sentence: “*This topography is consistent with findings in rats, in which the external lateral parabrachial subnucleus projects strongly to the anterior paraventricular thalamic nucleus (Pa), and less so to the middle and posterior paraventricular thalamic nucleus – (Pa)(Krout and Loewy, 2000).*” (Hsu and Price, 2009)

Relations: *nsubj*(projects-17, subnucleus-16), *det*(subnucleus-16, the-12), *amod*(subnucleus-16, external-13), *amod*(subnucleus-16, lateral-14), *nn*(subnucleus-16, parabrachial-15)

The candidate brain regions were returned in sorted order by their positions in the sentence:

the-12, external-13, lateral-14, parabrachial-15, subnucleus-16.

##### Special case for nsubj where the pattern keyword is in dobj

This specific case was an extension of the rule set described in the previous subsection for *nsubj* relations that had a pattern keyword in a Direct Object (dobj) relation. Our candidate brain region detection algorithm started by finding the pattern keyword in a *dobj* relation type. Then, the governor of the *dobj* relation was searched as the governor of a Nominal Subject (*nsubj*) relation. The dependent of the *nsubj* relation was taken as a candidate brain region. Differently from the other nsubj cases (described in the previous subsection), in this case, the *nsubj* relation did not need to contain the pattern keyword.

Additionally, this rule was extended to consider the modifiers of the nominal subject. Each dependent retrieved from a *nsubj* relation, was searched in the Adjectival Modifier (*amod*), Noun Compound Modifier (*nn*), and Prepositional Modifier (*prep*) relations as a governor. If such a relation was identified, the dependent of the relation was marked as a candidate brain region. Lastly, all identified candidate brain region words were returned in sorted order based on their sentence position information.

**Figure 5:**
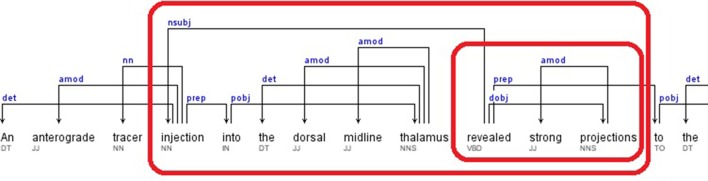
**Dependency Tree for the sentence: “*An anterograde tracer injection into the dorsal midline thalamus revealed strong projections to the accumbens nucleus, basal amygdala, lateral septum, and hypothalamus*”**.

A portion of the dependency tree of the following sample sentence is shown in Figure 5.

Sentence: “*An anterograde tracer injection into the dorsal midline thalamus revealed strong projections to the accumbens nucleus, basal amygdala, lateral septum, and hypothalamus.*” (Hsu and Price, 2009)

Relations: *dobj*(revealed-10, projections-12), *nsubj*(revealed-10, injection-4), *amod*(injection-4, anterograde-2), *nn*(injection-4, tracer-3), *prep_into*(injection-4, thalamus-9), *amod*(thalamus-9, dorsal-7), *amod*(thalamus-9, midline-8)

The candidate brain regions were returned in sorted order by their positions in the sentence:

anterograde-2, tracer-3, injection-4, dorsal-7, midline-8, thalamus-9.

##### Relations where the pattern keyword is a vmod

This group of rules first found the *vmod* relations where the pattern keyword was a dependent. In the next step, the complementary relations Adjectival Modifier (*amod*) or Noun Compound Modifier (*nn*) involving the governor of the identified *vmod* relation were retrieved. If any relation was found in this step, the dependent of the relation was retrieved as a candidate brain region. A sample sentence and the retrieved candidate brain regions are presented below.

Sentence: “*Here, we combined neuronal tract-tracing using the retrograde tracer cholera toxin b (CTb) with Fos expression to examine the effect of acute nicotine administration on orexin neurons projecting to the basal forebrain or PVT.*” (Pasumarthi and Fadel, 2008)

Relations: *vmod*(neurons-30, projecting-31), *nn*(neurons-30, orexin-29)

The candidate brain regions were returned in sorted order by their positions in the sentence:

orexin-29, neurons-30.

#### Relation decision

After the candidate generation phase (Section Candidate Generation Using NLP Techniques), the identified candidates were searched in the Brain Region Dictionary, in which a brain region (BR) was represented with its name, acronyms, and synonyms. A neuroanatomical relation was extracted, if at least two different brain region entities were matched in the dictionary, and one of them had the role of agent, whereas the other had the role of target. For the success of the dictionary matching process, we applied several steps as described below.

First we checked whether there was a full match between the agent/target and the dictionary entity (Step 1 in Table 4). If there was no match, this might have meant that the agent/target consisted of more than one brain region. Therefore, we split the text into strings from the conjunctions “and” and “or,” and the punctuation marks “comma” and “semicolon” (Step 2 and Step 3.a in Table 4; i.e., “the NAS, PFC, and amygdala” text was split as “NAS,” “PFC,” and “amygdala”). In addition, for each text string, there was a post-processing step, which removed some commonly used words like “of,” “the,” “area,” and “part.” If there was still no match for that text string, two more steps were applied. First, a substring search for this text string was done in all dictionary entities and the candidates were retrieved and secondly this text string was split into tokens from the spaces and then each token is searched in the brain region dictionary separately (Step 3.b in Table 4).

**Table 4:** **Relation decision phase using the dictionary on the annotated dataset**.

**Step**	**Candidate Agent/Target found by the Application**	**Annotated BRs in publication**	**Available Entities in Dictionary**	**Matching Type**
1	Text: “thalamus” Candidate Brain Regions: thalamus	Thalamus	thalamus	Full Match
2	Text: “the NAS, PFC and amygdala” Candidate Brain Regions: NAS PFC amygdala	NAS PFC Amygdala	NAS PFC Amygdala	Full Match
3	Text: “dorsal thalamus and SCN”	dorsal midline thalamus SCN	dorsal midline thalamus thalamus midline thalamus SCN	Full Match for SCN
3.a	Candidate Brain Regions: (tokenization by “and”) dorsal thalamus SCN			No match for dorsal thalamus
3.b	Candidate Brain Regions: (tokenization by space) dorsal thalamus			Partial match for thalamus

After finding the brain regions from the dictionary, only the longest version of the overlapping brain regions were selected. For example, if “thalamus” and “midline thalamus” are matched in the dictionary for a candidate brain region, then we selected “midline thalamus” as the extracted brain region. “Thalamus” was not selected, since it overlaps with “midline thalamus,” which is a longer match.

As the last step of relation extraction we defined whether the extracted brain regions were “full match” or “partial match” when compared with the annotated data set. If an extracted brain region matched only a part of the brain region in the annotated sentence, this was considered as a partial match. For example, assume that the application retrieved “thalamus” as a brain region and the manually annotated brain region text in the sentence was “dorsal midline thalamus.” In this case, the extracted brain region was considered as a partial match and the evaluation results were shown as “Lenient” in Section PVT case study, which meant that the extracted brain region might have been equal to or part of the annotated brain region.

### Evaluation approach

We used the precision, recall, and F-measure metrics to evaluate our relation extraction approach. The automatically extracted neuroanatomical connectivity relations (i.e., pairs of brain region entities) are compared with the manually annotated (gold standard) pairwise neuroanatomical relations. Precision is defined as the proportion of correctly retrieved neuroanatomical relations (i.e., true positives) to all the relations that the application retrieves (i.e., sum of true positives and false positives), whereas recall is defined as the proportion of correctly retrieved neuroanatomical relations (i.e., true positives) to all the neuroanatomical relations in the gold standard annotation (i.e., sum of true positives and false negatives). F-Measure is the harmonic mean of the precision and recall values.

#### Comparison with previous work using the whitetext corpus

We used the WhiteText corpus in order to compare our results with the previous studies (French et al., 2012, 2015; Richardet et al., 2015) that used the same data set processed with the abbreviation expansion algorithm of Schwartz and Hearst (2003). We processed the original abstracts obtained from PubMed using the same abbreviation expansion algorithm. In addition, French et al. (2012, 2015) and Richardet et al. (2015) evaluated their connectivity relation extraction methods by providing the gold standard brain region mentions as input. Therefore, instead of using our brain region dictionary, we used the gold standard brain region mentions provided in the annotations of the sentences in the WhiteText corpus. For instance, for the sample WhiteText annotation shown below, the candidate agents and targets identified by our approach were matched against “spinal trigeminal nucleus” and “cochlear nucleus” in the relation decision phase. The same matching strategy described in Section Relation Decision was used. Partial matches were also considered as correct.

<*entity id=“WhiteTextUnseenEval.d5917.s0.e0” text= “**spinal trigeminal nucleus**”./>*
<*entity id=“WhiteTextUnseenEval.d5917.s0.e1” text= “**cochlear nucleus**”. />*
<pair **interaction=“True”** e1= “WhiteText…s0.e1” e2=“WhiteText.s0.e0” />

The WhiteText corpus has been provided as two different data sets in time. In French et al. (2009, 2012), the first data set with 1377 annotated abstracts were shared, and then 1828 more abstracts were provided as the second data set of the WhiteText corpus (French et al., 2015). Richardet et al. (2015) also used the first data set during their research. In our approach (Linguistically Motivated Approach) we used the first data set while developing our system to improve the patterns and the NLP techniques that we applied, whereas the second data set with 1828 abstracts was only used as a test set. The second data set contains 2111 true connectivity relations. In these relations, there are also some interactions that include the same entities more than once in a sentence. Since we provided one pair for each sentence with the same entities in our application, we removed the redundant records from the evaluation. The total number of true-interactions that were used as gold standard was 1898.

#### Experiments for the PVT case study

For the PVT case study, we have two different evaluation sets.

In the first evaluation, connectivity relation extraction results are given for 14 full text publications, which are manually annotated by domain experts. Rather than using the manually annotated gold standard brain region mentions as done during the evaluation over the WhiteText corpus, we used our dictionary for identifying the brain regions that participate in the connectivity relations.

In the second evaluation, to provide automated extraction results on the PVT corpus, which consists of 558 publications, we executed our application on the abstracts of the 451 publications (the full text of which are not publicly available) and 107 full text publications (which are publicly available). We further used the output of this evaluation on connectivity graph generation.

#### Evaluation of directionality identification

The accuracy of our approach for finding the directionality of the connectivity relations was computed by considering the true positive relations extracted by our system. Accuracy was computed by calculating the proportion of true positive relations with correctly identified directionality to all true positive relations retrieved by our system.

## Results

### Evaluation on the whitetext corpus

Table 5 summarizes the results of our “linguistically motivated approach” and the results of the previous studies obtained on the WhiteText corpus. The corresponding data set information used by each study for evaluation is also shown.

**Table 5:** **Evaluation Results on the WhiteText Corpus of the proposed Linguistically Motivated Approach and the previous related studies**.

	**Precision (%)**	**Recall (%)**	**F-Measure (%)**
Linguistically Motivated Approach—2nd dataset (1828 abstracts)	76.94	14.59	24.53
(French et al., 2015)—2nd dataset (1828 abstracts)			
Shallow Linguistic Kernel (SLK)	51.00	67.00	57.92
Linguistically Motivated Approach—1st dataset (1377 abstracts)	75.60	17.31	28.17
(French et al., 2012)—1st dataset (1377 abstracts)			
Shallow Linguistic Kernel (SLK)	50.30	71.10	58.30
(Richardet et al., 2015)—1st dataset (1377 abstracts)			
Kernel (SLK)	60.00	68.00	64.00
Ruta Rules	72.00	12.00	21.00
Filter–Kernel	66.00	19.00	29.00
Kernel–Rules	81.00	10.00	18.00
Filter–Kernel–Rules	82.00	7.00	12.00

The total number of true-interactions that were used as gold standard in the WhiteText corpus test set (2nd data set) was 1898. We extracted 360 relations by using our application and 277 of these relations were true positives, whereas we misinterpreted 83 of these relations. Overall, the precision on the test set was 76.94% with a recall level of 14.59%. The only previous study that used the same data set is the study of French et al. (2015), which obtained 51% precision and 67% recall by using the Shallow Linguistic Kernel (SLK) originally proposed for protein interaction extraction by Giuliano et al. (2006). As one of the aims of our study, we achieved higher precision by using a knowledge-based approach compared to the kernel-based machine learning approach.

Additionally, Table 5 shows the results for the WhiteText corpus first data set, which contains 1377 abstracts and 3097 relations. Our application extracted 709 relations, where we had 536 true-positives, and 173 false-positives. This evaluation corresponded to 75.60% precision and 17.31% recall. Similarly to the WhiteText corpus second data set, the study of French et al. (2012) obtained 70.10% recall and 50.30% precision by using the SLK. On the same data set, Richardet et al. (2015) provided their results for different combination of Kernel, Filters and Rules. In Table 5, Kernel represents the machine learning model (i.e., the Shallow Linguistic Kernel), the Ruta rules are the ones that are manually crafted on the Apache UIMA Ruta workbench (Kluegl et al., 2014), and filters are the custom filters like discarding some sentences. By applying the kernel-based approach only, they improved the results of French et al. and obtained 60% precision with %68 recall. Their rule-based approach obtained 72% precision and 12% recall. Being a rule-based method targeting higher precision at the expense of recall, the rule-based method of Richardet et al. (2015) is the most similar one to ours. The rules in Richardet et al. (2015) are defined over the surface forms of the sentences. On the other hand, our rules utilize the syntactic and dependency parse trees of sentences leading to both higher precision and higher recall values. Richardet et al. (2015) achieved their highest precision of 82% by combining their three approaches (i.e., kernel, filter, and rules). However, this lowered recall to 7%.

Using a knowledge-based approach came with more accurate results with the cost of missed relations when it is compared with the semi-automated or fully automated machine learning techniques. Therefore, comparing our approach with the Kernel results of French et al. (2012, 2015) and Richardet et al. (2015), the precision we obtained was higher, whereas the recall was lower. On the other hand, comparing with rule based approach of Richardet et al. (2015), we achieved higher recall, since we had more fine-grained rules at the linguistic level.

### PVT case study

A particular point of interest and a motivating factor in our undertaking the present study was due to a bottom-up view of depression proposed by one of us (Canbeyli, 2010, 2013). Briefly, it was proposed that mood and depressive symptoms can be modulated by varying the intensity, duration and quality of stimulation by means of sensory input via visual, auditory, taste and olfactory systems, among others, as well as physical exercise. This bottom-up approach, in contradistinction to the more established account of depression and its therapies by top-down processes, is able to integrate a large body of evidence from studies that have manipulated depression by sensorimotor modulation in animal models of mood and depression and offers a new avenue of potential treatments for depression in humans. Canbeyli (2013) proposed a circuitry for the integration of bottom-up sensorimotor peripheral input to the neurocircuitry underlying depression in humans and animals with “top-down”—potentially more cognitive influences—from the neocortex. The amygdala in particular was proposed as a key element in the nexus of the top-down and bottom-up processes. While the amygdaloid complex is a critical component of the neurocircuitry of depression, it is remarkable that the PVT, particularly with its connections to lower brainstem structures involved in visceromotor input and its connections to the amygdala, the infra- and pre-limbic cortices as well as the subgenual cingulated gyrus area, is also in a position to integrate the bottom-up sensorimotor influences. As the PVT connectivity graph and the following discussion will show, PVT may be a new target of research in mood assessment.

#### Evaluation on the annotated PVT corpus

For the evaluation of the PVT case study, we used the 14 manually annotated full texts, which were PVT specific publications.

As the output of the Relation Extraction phase (Section Neuroanatomical Relation Extraction), we generated the candidate relation pairs constructed of the agents and targets. The brain region dictionary we created (Section Creation of a Brain Region Dictionary) was used to validate the existence of brain region entities in the texts of the agents and targets. Therefore, the impact of a comprehensive dictionary was very high on the accuracy of the evaluation results.

The manually annotated data set of PVT from the 14 publications used in the present study contained 322 relations: 97 of these relations did not have any of our predefined patterns (Table 3) in their corresponding sentences. Therefore, they were already missed, since the corresponding sentences were not selected as candidates for further processing. In the light of this information, the maximum level of recall that our approach could reach was 69.88%.

Using NLP techniques, our application extracted 161 relation candidates out of 225 “pattern-including” relations. When we compared each relation candidate with the annotated data set, the number of full matches was 107 and the number of partial matches was 15, whereas the number of incorrect predictions was 20. For the remaining 19 relation candidates, we evaluated the results in two different ways. These 19 candidates included the agents and the targets and were matching with the brain region entities in the brain region dictionary. This meant that we hit a relation with correct brain regions; therefore we evaluated these values as full or partial matches. We shared these results as NLP-based results in Table 6. On the other hand, during the annotation process, these relations were found to be too generic or ambiguous and eliminated depending on the sentence structure. In this second approach they were considered as incorrect predictions and were given as part of Strict and Lenient evaluations.

**Table 6:** **PVT case study evaluation**.

	**Precision (%)**	**Recall (%)**	**F-Measure (%)**
Strict (Full Match)	66.43	33.23	44.30
Lenient (Full Match + Partial Match)	75.78	37.89	50.52
NLP-based	87.58	43.79	58.39

The following sentence contained three of these 19 relations. Our application retrieved the relation candidates “PVT”-“PFC,” “PVT”-“NAS,” and “PVT”-“AMG” and they are likely to refer to a relation. However, these relations were considered either too generic or ambiguous, and therefore, have not been manually annotated in the data set.

“*.., it appears likely that there are no substantial differences in the degree to which stress activates PVT neurons that **innervate** the PFC, NAS and AMG.”* (Bubser and Deutch, 1999)

Actually, this is one of the core points that we would like to highlight with automated relation extraction. Using different techniques, we can automatically extract brain region relations, but this is still an input for further evaluation and domain knowledge is crucial to turn this input to valuable information. We consider this NLP-based evaluation as also valuable and share it in addition to the Strict and Lenient results. Table 6 shows these evaluation results by classifying them as Strict Comparison, which is the full-match of brain regions from the dictionary, Lenient Comparison, which is the full matches and partial matches of the brain regions, and lastly NLP-based comparison, which additionally includes the true-positive relations that the application finds, but not annotated by domain experts.

When we compared and evaluated the WhiteText and PVT corpora, we reached two conclusions. Firstly, recall value was higher with the PVT corpus, and the main reason was the percentage of the sentences that we could match with the patterns. For the WhiteText corpus, the maximum recall that we could reach was 57.7%, whereas for PVT annotated corpus it was 69.88%. Thus, the PVT corpus contained more relations aligned with the patterns.

Secondly, the precision values of the patterns were similar across the two data sets. Although the patterns were tuned based on the WhiteText corpus, they could effectively be applied to other data sets in this domain with precision levels of at least 70–75%.

Lastly, the total number of document-level unique relations was computed by eliminating the duplicate relations occurring in the same document so that a pair of brain regions was extracted only once from the document. Out of 322 relations in the 14 annotated papers, the total number of relations that are unique at document-level was 237. Only 7 of these 237 relations were in the abstract part of the publications, which meant that only 3% of the relations were available in the abstracts within this corpus. Using full text publications instead of abstracts mostly assured to obtain more relations to be extracted. A strength of our system is that it obtained the same success level on full text documents as well as on abstracts.

The PubMed IDs of the 14 annotated PVT papers and the annotated sentences are shared as Supplementary Materials. Considering that some of the publications are not publicly available, the publications are not fully provided.

#### Full PVT corpus and connectivity graph

We ran our application for the data set which consisted of 558 publications (451 abstracts and 107 full text publications) and 811 relations were extracted from this corpus including 343 different brain regions. Further analysis on the relations showed that PVT was the target of 75 relations, and the source of 92 relations. Table 7 shows the top five brain regions with the highest number of total relations and Table 8 shows the five most frequent relations that are extracted from the PVT data set. It is not surprising that PVT is the most highly connected brain region in this corpus, since the corpus was created from publications relevant to PVT. The other highly connected brain regions in this PVT specific corpus are locus coeruleus, nucleus accumbens, suprachiasmatic nucleus, and amygdala. Besides nucleus accumbens and amygdala, prefrontal cortex and medial prefrontal cortex are other brain regions frequently occurring as targets in a PVT connectivity relation in the literature. On the other hand, suprachiasmatic nucleus occurs more frequently as an agent.

**Table 7:** **Top brain regions as agent or target in a relation**.

**Brain Region**	**Agent**	**Target**	**Total**
PVT	92	75	167
Locus coeruleus	39	23	62
Nucleus accumbens	8	47	55
Suprachiasmatic nucleus	30	18	48
Amygdala	10	29	39

**Table 8:** **Top Relations that are automatically extracted from PVT Corpus**.

**Agent**	**Target**	**Number of Relations**
PVT	Nucleus accumbens	23
PVT	Prefrontal cortex	13
Suprachiasmatic nucleus	PVT	10
PVT	Amygdala	8
PVT	Medial prefrontal cortex	6

**Figure 6:**
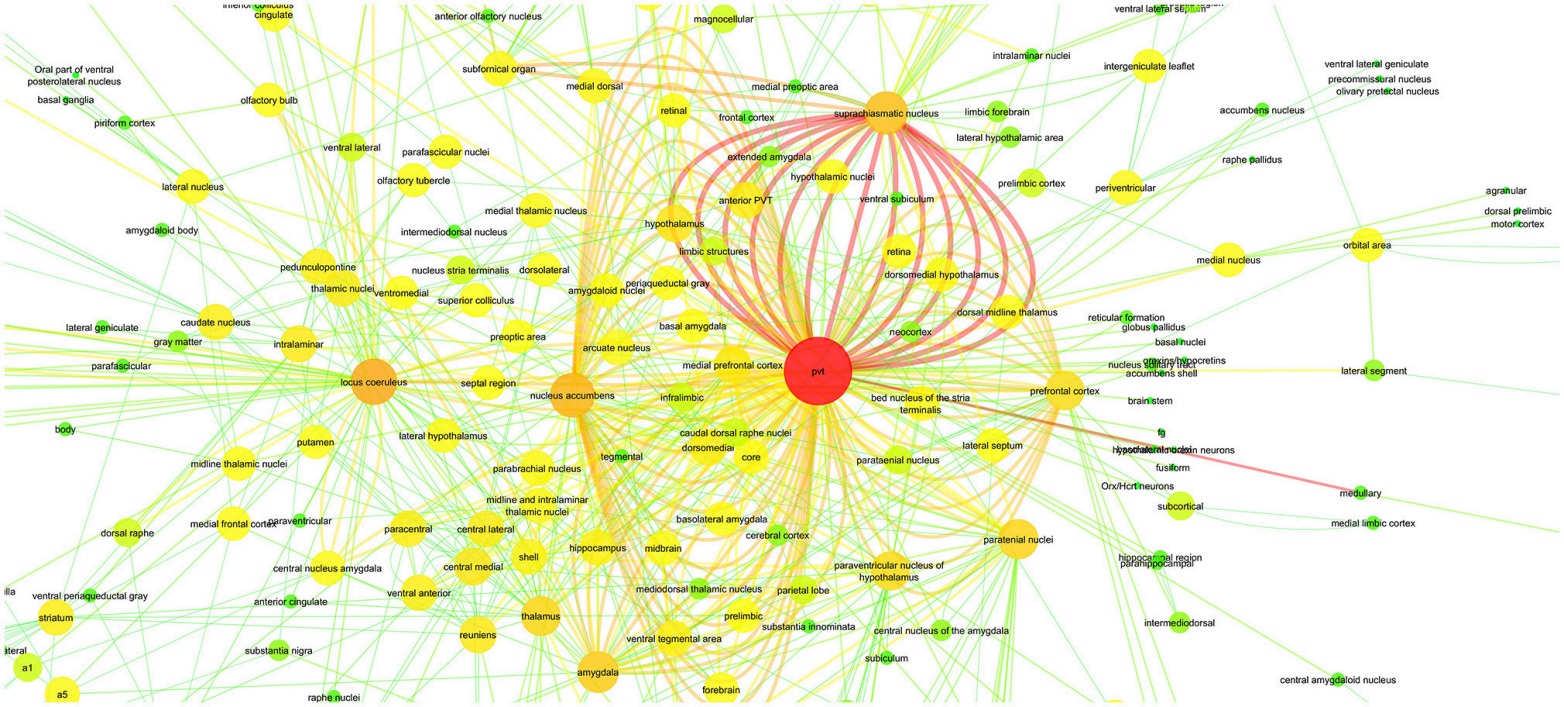
**PVT Connectivity Graph: With Color Mapping of edge count on nodes and edge betweenness on edges**.

In Figure 6, we applied these 811 relations to a connectivity network graph. The brain regions are defined as nodes and the edges between them represent the literature-mined connectivity relations. For each connectivity mention extracted between a pair of brain regions from the literature, an edge is added between the corresponding nodes. The higher the number of edges is between two nodes in the graph, the more connectivity mentions between the corresponding pair of brain regions were extracted from the publications. We use a color map where green and yellow represent low edge counts for a node, whereas orange and red are used for higher edge counts for a node. The nodes of the graph grew larger according to the edge count. Similarly, for the edge color mapping we used edge betweenness. The edge betweenness of an edge is defined as the number of the shortest paths between pairs of nodes that run along it (Girvan and Newman, 2002). High edge betweenness score means that if this edge is removed, it will have a high impact on the connections between the nodes.

While creating the graph, the agents and the targets were matched with the unique entities in the dictionary.

Various versions of the connectivity graph (with the arrows showing the direction or with different network analysis styles) are given as Supplementary Materials.

### Directionality of the relations

One of the contributions of our research to existing works was to define the direction of the relations.

As mentioned in the Relation Decision section, we defined a rule for each pattern that determines the direction of the relation. During the test phase of the WhiteText and PVT corpora, in addition to agent and targets we also added the directionality information as the output. The accuracy of the directionality prediction approach is shown in Table 9.

**Table 9:** **Accuracy of the direction prediction for each corpus**.

**Corpus**	**Number of True Relations in the Corpus**	**Number of Relations Examined for Direction**	**Number of Correctly Identified Directions**	**Accuracy (%)**
*WhiteText* corpus	1898	277	277	100.00
PVT Corpus (14 annotated publications)	322	122	119	97.54

From the second dataset of the WhiteText corpus, we obtained 277 true positive relations out of 360. In addition to extracting 277 relations correctly, the accuracy of the directions was calculated as 100%, which is also validated by one of the authors (RC)^4^. Since the remaining 83 false positive relations were already misclassified, we did not check their directionalities. The results also showed that 205 of these directions were from the first brain region mention to second brain region mention, and 72 of the predicted directions were from the second one to the first one. So, a majority classifier baseline classifying each direction from the first brain region mention to the second would obtain an accuracy of 74%, which is considerably lower than the accuracy achieved by our approach.

For the annotated PVT corpus, 122 out of 322 relations were retrieved. When we evaluated the directionality of these relations, 119 out of 122 were predicted correctly which corresponds to an accuracy of 97.54%. As shown in Table 7, PVT, locus coeruleus, and the suprachiasmatic nucleus were the main agents initiating the projections. On the other hand, PVT, nucleus accumbens, and amygdala were the main targets in these connectivities.

## Discussion

A major aim of the present study was to provide a new approach in text mining to chart out neuroanatomical connections of a specific brain structure. We have presented a linguistically motivated approach to extract neuroanatomical connectivity relations from scientific publications by using NLP techniques. Our approach leverages the constituency and dependency parse trees of sentences and defines the agents and the targets by also providing the directionality of the relations.

The strength of our approach comes from the patterns and rules that are defined over the parse trees of the sentences. The selection criteria for the patterns heavily depend on the individual success of each pattern to lead to a relation. We use the patterns to identify candidate sentences for further processing and relation extraction. A limitation of our approach is that only relations from sentences that match one of our predefined patterns can be extracted. On the other hand, whenever a pattern is found in a sentence, it is very likely that a relation extracted after further processing is correct. Therefore, our expectation from the present study was to obtain high precision and low recall values. We preferred to have a target of at least 60% precision level for each pattern, and as a consequence, the maximum recall value that our application could reach was approximately 70% (on the PVT data set). It is up to the researchers to define the optimum level for their evaluations. In this study, our goal was to design a high precision system so that many false positive relations are not included in the brain region connectivity graph, which could lead to incorrect interpretations.

Most previous studies on connectivity extraction among brain regions from text used machine learning based methods originally proposed for extracting protein-protein interactions. French et al. (2012) evaluated seven kernel functions for the task of brain region connectivity extraction, which have been benchmarked for protein-protein interaction extraction by Tikk et al. (2010). On the WhiteText corpus all-paths graph kernel (Airola et al., 2008) and k-band shortest path spectrum kernel (Tikk et al., 2010), which make use of the dependency parses of sentences, obtained similar performances to the Shallow Linguistic Kernel (SLK) (Giuliano et al., 2006), which only uses shallow linguistic information including surface forms of words, word lemmas, and part-of-speech tags. The other evaluated kernel functions, namely subset tree kernel (Collins and Duffy, 2001), partial tree kernel (Moschitti, 2006), spectrum tree kernel (Kuboyama et al., 2007), and subtree kernel (Vishwanathan and Smola, 2004) are based on the constituency parses of sentence. These functions obtained significantly lower scores than the first three kernel functions both in terms of precision (lower than 45%) and recall (lower than 26%) (French et al., 2012). These results are in agreement with the ones reported for the task of protein-protein interaction extraction by Tikk et al. (2010), where SLK obtained 47.5% precision and 54.5% recall over the commonly used AIMED data set, and performed similarly to the dependency tree based kernel functions, which achieved superior performance compared to the constituency parse tree based kernel functions. The results by Tikk et al. (2010) and French et al. (2012) do not reveal a clear strength in using dependency and constituency analysis in a kernel based supervised machine learning set-up, since SLK obtains better or similar performance compared to the kernel functions that are based on deeper syntactic analysis. On the other hand, our study shows that using dependency and constituency parsing improves performance in a rule-based set-up for brain region connectivity extraction. Richardet et al. (2015) extended the study by French et al. (2012), with Ruta rules (Kluegl et al., 2014) and custom filters. Ruta rules, which were manually crafted on the Apache UIMA Ruta workbench (Kluegl et al., 2014) according to the structures of the sentences, obtained precision of 72.00% and recall of 12.00% on the WhiteText corpus. Our rule-based approach, which utilizes deeper linguistic analysis of sentences, achieved higher precision (75.60%) and higher recall (17.31%) than the rule-based approach of Richardet et al. (2015), and higher precision than the kernel based machine-learning methods in French et al. (2012, 2015) and Richardet et al. (2015) at the cost of lower recall. Although Richardet et al. (2015) achieved the highest precision of 82.00% by combining the machine learning approach with rules and filters, this resulted in a significantly lower recall level of 7%.

Additionally, by using the predefined patterns to find the agent and the target, we were able to make a contribution on a missing feature of prior work on relation extraction: directionality of the relation. According to the grammatical structure of the sentences and the pattern usages, we identified the relation directionality between the brain regions and the overall accuracy of extracted directions was more than 97%.

In the PVT case study, we used a dictionary-based approach while extracting the brain regions from publications. It is known that in the neuroscience literature brain region entities are not used in a unique and standardized way. There are several different names of each brain region and the corresponding abbreviations may vary. Using brain region mentions directly without normalizing them to canonical brain region names would result in redundant entities (nodes) that referred to the same brain region in the connectivity graph. By using a dictionary, we accepted the possible loss on finding all the brain regions from the texts, but on the other hand we leveraged the dictionary usage on the connectivity graph by providing canonical names for the brain regions.

A decision point for us was whether to use the existing ontologies or to create our own dictionary. Before constructing the dictionary, we investigated the existing brain ontologies. Brain Architecture Management System (BAMS) (Bota and Swanson, 2008) is an ontology that includes brain regions and their relations for rats. Neuroscience Information Framework Standard Ontology (Bug et al., 2008) and Textpresso (Muller et al., 2008) are also comprehensive resources on the neuroscience domain. These ontologies are very helpful to have a standard consistent terminology of the brain regions with their acronyms and synonyms. A disadvantage of these ontologies is that they are not specifically defined for text mining purposes. Relevant publications do not commonly use brain region names as they are referred to in these ontologies. For example, while most of the brain regions in these ontologies are given with “nucleus” attached to the structures, in the publications the authors can omit the term “nucleus” (e.g., “dorsomedial” is used instead of “dorsomedial nucleus”). Another example is the usage of “caudal DR” in the publications instead of “caudal dorsal raphe,” which is the form that occurs in most available ontologies. Secondly, authors may prefer to use different acronyms instead of the widely used acronyms of the brain regions. For example, BrainInfo portal^5^ which contains the NeuroNames knowledgebase, uses “PV” and “PVT” as acronyms of paraventricular nucleus of the thalamus, whereas in some publications “Pa” is used as an acronym for the same brain region Hsu and Price (2009). French and Pavlidis (2012) and Richardet et al. (2015) showed that creating dictionaries by expanding the brain region names in the existing ontologies by including synonyms gathered from the literature improves recall in named entity recognition and normalization. We followed a similar approach to these previous studies and created a brain region dictionary by including synonyms that we manually compiled from the literature for the brain region names in the NeuroNames ontology (Bowden and Dubach, 2003) and NeuroLex (Larson and Martone, 2013). Since we needed to obtain the anatomical directions during the text mining process, we created the brain region entities in the dictionary with the direction information such as anterior, posterior, ventral, dorsal, rostral, caudal, etc.

During the relation extraction phase, we faced several difficulties. One was related to the WhiteText corpus. This manually annotated corpus was considered as gold standard for the first evaluation phase of our research. Since this corpus is enhanced with the abbreviation expansion algorithm, we also needed to use the same approach. Schwartz and Hearst Abbreviation Expansion Algorithm (Schwartz and Hearst, 2003) is used for this purpose and it requires the replacement of the short forms of the abbreviations with their long forms. The short form is also added right after the long form. We skipped this step on the PVT case study, since the abbreviations were already included as part of the brain region dictionary under the rubric of acronyms. Another challenge while extracting the relations was the ambiguity when brain regions are used in the text with conjunctions (e.g., “dorsal and ventral cortex” or “basolateral and basomedial nuclei of the amygdala”). We initially decided to evaluate these phrases as one brain region entity, since the WhiteText corpus considered such phrases as one brain region mention. For the PVT corpus, we needed to remove the conjunction and create two different brain region entities from these mentions. After the implementation of this phase, we noticed that the overall precision was reduced due to false positives, hence, we kept ambiguity resolution as a project for future work.

An additional aim of the present study was to provide by means of a connectivity graph an overview of the neuroanatomical connectivity relations of PVT that may suggest potentially new functions for the midline thalamic structure. As demonstrated in Figure 6, PVT has far reaching direct connectivity with a large number of brainstem, subcortical and cortical structures. These neuroanatomical connections have yet to be adequately interpreted in terms of potential functions that may be served by subcircuits involving a more restricted number of PVT connections. Nevertheless, there is a growing realization that the PVT is not merely a component of a general behavioral arousal mechanism or a stress circuitry (Bubser and Deutch, 1999; Vertes et al., 2015), but is likely to be critically involved in more specific functions. Our PVT case study listed SCN (suprachiasmatic nucleus) and nucleus accumbens among the brain regions with central roles in a PVT specific connectivity graph (Tables 7, 8). Recent studies in the literature support this hypothesis by suggesting new functions for PVT based on its connectivity. It has been suggested that PVT may be an important factor in sleep/wake cycles because it is connected with hypothalamic structures such as the SCN (suprachiasmatic nucleus) and dorsomedial hypothalamus and receives strong orexigenic projections from the hypothalamus (Colavito et al., 2015). Furthermore, due to its prominent relationship with the nucleus accumbens, PVT has been investigated in connection with reward mechanisms and drug addiction (Matzeu et al., 2014).

In the light of the vast connectivity uncovered by our present study, we hope that there may be more interest in delineating neuroanatomical subcircuits involving the PVT as potential substrates for various functions. Toward that goal, we hereby propose in outline form a PVT circuitry that we hope to elucidate in a future article that may be underlying a mood modulatory mechanism. Briefly, our analysis of PVT connections has uncovered a strong connectivity between the PVT and several structures known to be involved in mood and depression in both humans and animals. As demonstrated in Tables 7, 8 and Figure 6, PVT has its strongest connection (i.e., highest number of connectivity mentions in the literature) with SCN. It is also connected with the nucleus accumbens, the amygdaloid complex and the extended amygdala that includes the bed nucleus of the stria terminalis (BNST) and the ventromedial prefrontal cortex. Along with other functions that they may share, these structures are also involved in mood and depression especially as indicated by studies on animal models of depression. Thus, depression as measured by forced swimming in rats is reduced with SCN (Tataroğlu et al., 2004), aggravated by BNST lesions (Schulz and Canbeyli, 2000; Pezuk et al., 2008), while stimulation of the ventromedial prefrontal cortex reduces depression in both humans (Koenigs and Grafman, 2009) and rats (Hamani et al., 2010). Animal studies also indicate that disruption of the nucleus accumbens results in anhedonia, which is a major symptom of depression in both humans and animals (Willner, 1990; Russo and Nestler, 2013). Despite such evidence, there is a paucity of studies that have directly addressed the issue of PVT involvement in depression. In the only relevant study so far, Zhu et al. (2011) have shown that co-increase in c-fos positive neurons in the PVT and the central nucleus of the amygdala (CE) in rats subsequent to forced swimming rats may indicate that PVT neurons are engaged in acute depressive events.

In our study, we have focused on automated connectivity relation extraction of brain regions in the neuroscience domain. Hence, our defined patterns and rules might not be generic enough to be used in other domains such as Protein-Protein and Gene-Disease interactions. This is considered as a possible future work. Similarly to previous studies on brain region connectivity extraction (French et al., 2012, 2015; Richardet et al., 2015), our patterns and rules operate on sentence-level. Relations that span multiple sentences are not tackled. French et al. (2012) showed that the description of around 27% of the connectivity relations cross sentence boundaries in the WhiteText corpus. Addressing the extraction of such relations by utilizing anaphora resolution techniques is an interesting and useful future direction for research. Additionally, the current research identifies only the neuroanatomical connectivity relations of the brain regions (circuitry). As future work, the chemical connections between brain regions (neurotransmitters) and the functional connections (by the attributed cognitive function of the relation) will be our focus of interest.

## Author contributions

EG, carried out the computational studies, performed the implementations of the algorithms, and participated in the design of the study, analysis of the results, and drafting of the manuscript. AÖ, participated in the design of the study, analysis of the results, and drafting of the manuscript. RC, participated in the design of the study, analysis of the results, annotation of the data and drafting of the manuscript. All authors read and approved the manuscript.

### Conflict of interest statement

The authors declare that the research was conducted in the absence of any commercial or financial relationships that could be construed as a potential conflict of interest.

## Footnotes

^1^https://github.com/erincgokdeniz/relation_extraction/tree/master/PVTResearch/supplementary.

^2^Supplementary Materials can be found at: https://github.com/erincgokdeniz/relation_extraction/tree/master/PVTResearch/supplementary.

^3^Stanford Parser Dependencies Manual can be found at: http://nlp.stanford.edu/software/dependencies_manual.pdf.

^4^The manually annotated directions for the true positive relations extracted from the WhiteText and PVT corpora are available as supplementary files as well as in Github repository: https://github.com/erincgokdeniz/relation_extraction/tree/master/PVTResearch/supplementary.

^5^http://www.braininfo.org,

## Supplementary material

Data Sheet 1 **Supplementary Material documentation**.

Data Sheet 2 **Patterns**.

Data Sheet 3 **PVT Corpus—PubMed IDs of Abstracts**.

Data Sheet 4 **PVT Corpus—PubMed IDs of Full Text Publications**.

Data Sheet 5 **SPVT Corpus—PubMed IDs of Annotated Papers**.

Data Sheet 6 **Annotations and PMIDs of Annotated Papers**.

Data Sheet 7 **PVT—Direction Annotated**.

Data Sheet 8 **Whitetext—Direction Annotated**.

Data Sheet 9 **Brain Region Dictionary**.

Image 1 **Connectivity Graph with Directions**.
