# Supplementary material for: Automated Neuroanatomical Relation Extraction: A Linguistically Motivated Approach with a PVT Connectivity Graph Case Study
Source: Front Neuroinform. 2016 Sep 21;10:39. doi: 10.3389/fninf.2016.00039 (PMC5030238; doi:10.3389/fninf.2016.00039)
Supplement: Data Sheet 1 — Supplementary Material documentation. [file DataSheet1.docx]

Supplementary Material

Automated Neuroanatomical Relation Extraction: A Linguistically Motivated Approach with PVT Connectivity Graph Case Study

Erinç Gökdeniz*, Arzucan Özgür*, Reşit Canbeyli*

*** Correspondence:** Corresponding Author, [erincgokdeniz@gmail.com](mailto:erincgokdeniz@gmail.com), [arzucan.ozgur@boun.edu.tr](mailto:arzucan.ozgur@boun.edu.tr) , [canbeyli@boun.edu.tr](mailto:canbeyli@boun.edu.tr)

# Supplementary Data

Following supplementary data is provided with this document. They are submitted as separate files.

- Patterns (Data Sheet 2.docx)
- Datasets
  - PVT Corpus - PubMed IDs of Abstracts (Data Sheet 3.csv)
  - PVT Corpus - PubMed IDs of Full Text Publications (Data Sheet 4.csv)
  - PVT Corpus - PubMed IDs of Annotated Papers (Data Sheet 5.csv)
- Annotated sentences for PVT Corpus (Data Sheet 6.xlsx)
- Annotated directions for the PVT Corpus (Data Sheet 7.xlsx)
- Annotated directions for the WhiteText Corpus (Data Sheet 8.xlsx)
- Brain Region Dictionary (Data Sheet 9.csv)
- Connectivity Graph with Directions (Image 1.jpeg)
