# Supplementary material for: Automated Neuroanatomical Relation Extraction: A Linguistically Motivated Approach with a PVT Connectivity Graph Case Study
Source: Front Neuroinform. 2016 Sep 21;10:39. doi: 10.3389/fninf.2016.00039 (PMC5030238; doi:10.3389/fninf.2016.00039)
Supplement: Data Sheet 2 — Patterns. [file DataSheet2.docx]

## Patterns :

| Pattern Name | Regular Expression |
| --- | --- |
| innervate | (?i)innervat(e\|es\|ing){1} |
| innervation of | (?i)innervation(s){0,1} of |
| projection to | (?i)projection(s){0,1} to |
| projection to from | (?i)projection(s){0,1} to ((\\w+)\\s){0,8} from |
| projection of | (?i)projection(s){0,1} of |
| projection target of | (?i)projection target(s){0,1} of |
| projection from | (?i)(the ){0,1}projection(s){0,1} from |
| projection from to | (?i)projection(s){0,1} from ((\\w+)\\s){0,8} to |
| project to | (?i)project(ing\|s\|ed){0,1} ((\w)* ){0,2}to |
| project into | (?i)project(ing\|s\|ed){0,1} ((\w)* ){0,2}into |
| project from | (?i)project(ing\|s\|ed){0,1} from |
| project from to | (?i)project(s\|ed\|ing){0,1} from ((\\w+)\\s){0,8} to |
| receive input from | (?i)receiv(e\|es\|ing\|ed){0,1} ((\w)* ){0,4}input(s){0,1} ((\w)* ){0,3}(from) |
| receive fiber from | (?i)receiv(e\|es\|ing\|ed){0,1} ((\w)* ){0,4}fiber(s){0,1} ((\w)* ){0,3}(from) |
| receive innervation from | (?i)receiv(e\|es\|ing\|ed){0,1} ((\w)* ){0,4}innervation(s){0,1} ((\w)* ){0,3}(from) |
| receive [ae]fferent from | (?i)receiv(e\|es\|ing\|ed){0,1} ((\w)* ){0,4}[ae]fferent(s){0,1} ((\w)* ){0,3}(from) |
| send via to | (?i)(((sen(d\|ds\|ding\|t)) ((\w)* )*via ((\w)* )*to)) |
| send from | (?i)(((sen(d\|ds\|ding\|t)) from ((\w)* )*to)) |
| send to | (?i)(sen(d\|ds\|ding\|t)) ((\w)* ){0,2}to |
| travelling from to | (?i)travel(s\|ling){0,1} ((\w)* ){0,2}from ((\w)* ){0,5}to |
| travel through | (?i)travel(s\|ling){0,1} ((\w)* )*through |
| exit through | (?i)exit(s\|ing){0,1} ((\w)* )*through |
| exit from | (?i)exit(s\|ing){0,1} ((\w)* )*from |
