# Supplementary figures and images for: Automated Neuroanatomical Relation Extraction: A Linguistically Motivated Approach with a PVT Connectivity Graph Case Study
Source: Front Neuroinform. 2016 Sep 21;10:39. doi: 10.3389/fninf.2016.00039 (PMC5030238; doi:10.3389/fninf.2016.00039)

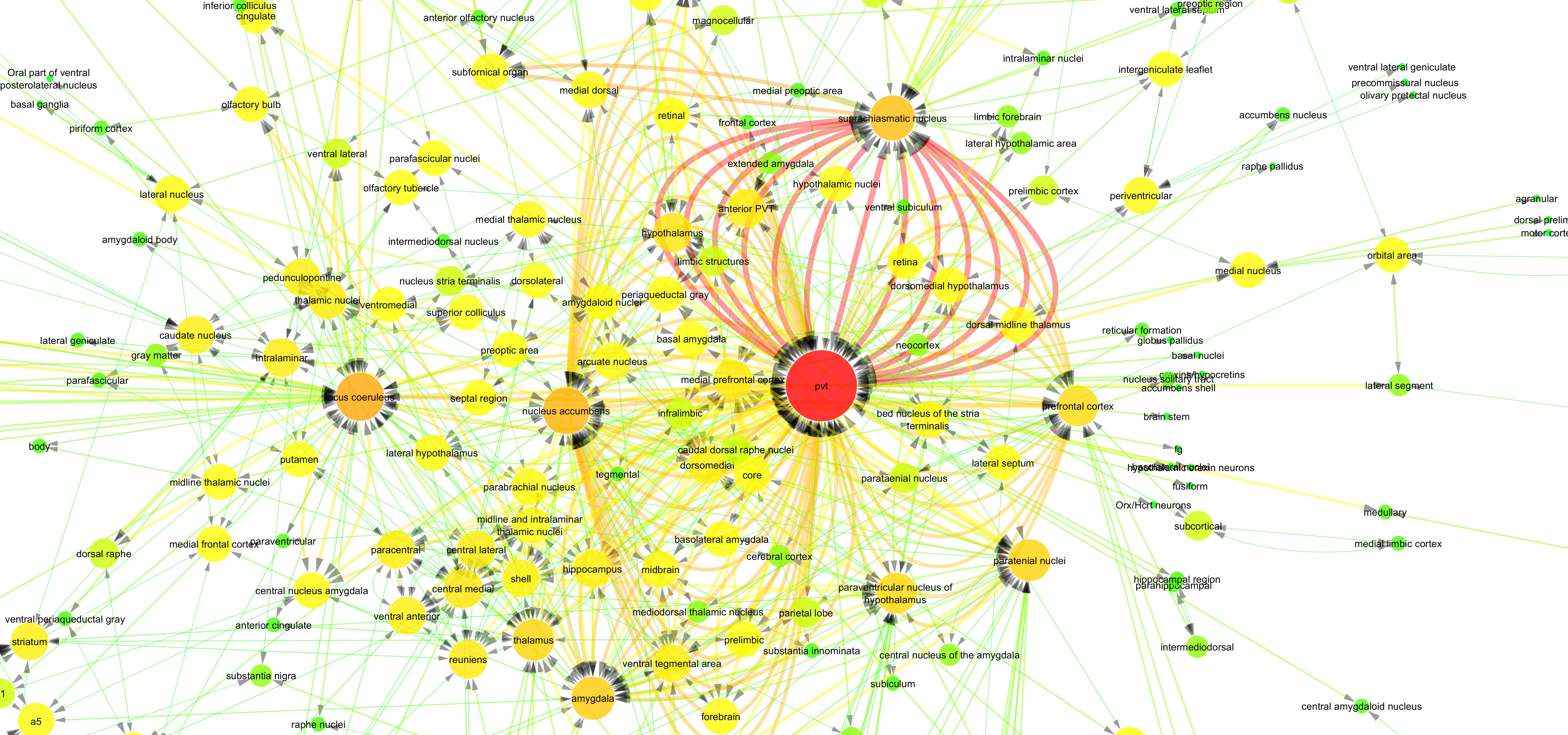

Supplement: Image 1 — Connectivity Graph with Directions. [file Image1.jpeg]
